# Brain sugar consumption during neuronal activation detected by CEST functional MRI at ultra-high magnetic fields

**DOI:** 10.1038/s41598-019-40986-9

**Published:** 2019-03-14

**Authors:** Tangi Roussel, Lucio Frydman, Denis Le Bihan, Luisa Ciobanu

**Affiliations:** 1 NeuroSpin, Commissariat à l’Energie Atomique et aux Energies Alternatives, Univerisité Paris-Saclay, Gif-sur-Yvette, France; 2 Department of Chemical and Biological Physics, Weizmann Institute of Science, Rehovot, Israel

## Abstract

Blood oxygenation level dependent (BOLD) functional magnetic resonance imaging (fMRI) indirectly measures brain activity based on neurovascular coupling, a reporter that limits both the spatial and temporal resolution of the technique as well as the cellular and metabolic specificity. Emerging methods using functional spectroscopy (fMRS) and diffusion-weighted fMRI suggest that metabolic and structural modifications are also taking place in the activated cells. This paper explores an alternative metabolic imaging approach based on Chemical Exchange Saturation Transfer (CEST) to assess potential metabolic changes induced by neuronal stimulation in rat brains at 17.2 T. An optimized CEST-fMRI data acquisition and processing protocol was developed and used to experimentally assess the feasibility of glucoCEST-based fMRI. Images acquired under glucose-sensitizing conditions showed a substantial negative contrast that highlighted the same brain regions as those activated with BOLD-fMRI. We ascribe this novel fMRI contrast to CEST’s ability to monitor changes in the local concentration of glucose, a metabolite closely coupled to neuronal activity. Our findings are in good agreement with literature employing other modalities. The use of CEST-based techniques for fMRI is not limited to glucose detection; other metabolic pathways involved in neuronal activation could be potentially probed. Moreover, being non invasive, it is conceivable that the same approach can be used for human studies.

## Introduction

Blood Oxygenation Level Dependent functional imaging (BOLD fMRI) is one of the keystones of modern neuroimaging^1–3^. BOLD imaging allows the indirect measurement of brain activity by detecting changes in the blood oxygenation level and relying therefore on the neurovascular coupling as a reporter^4^. Emerging methods such as functional MR spectroscopy (fMRS), vascular-space occupancy fMRI, and diffusion-weighted fMRI, are opening alternative ways of studying neuronal activation by detecting changes in metabolism^5^, blood volume^6^ and water diffusion properties^7^. fMRS studies, in particular, reported changes in glucose^8,9^ metabolism during activation, highlighting important metabolic changes linked to neuronal activity. Chemical Exchange Saturation Transfer (CEST) is another imaging technique with expanding uses as metabolic reporter, yielding a magnified contrast for selected compounds possessing labile hydrogens capable of undergoing chemical exchanges with the protons of water^10^. Endogenous CEST contrast arises from the changes that the water signal intensity will exhibit when selectively irradiating these exchangeable metabolic sites, which can be targeted depending on the irradiation frequency and amplitude. While existing CEST methods are typically less selective than MRS techniques, CEST can be employed to detect and image specific metabolites with sensitivities that can exceed, under some conditions, those of direct spectroscopic detection. CEST metabolic imaging has been reported for fundamental metabolites such as glucose^11^, glutamate^12^, lactate^13^ and GABA^14^. Comparing the sensitivities of CEST and MRS experiments is not straightforward, as these will depend on many instrumental and physiological parameters such as pH, temperature, B_0_ and B_1_ inhomogeneities, spectral overlapping and water suppression efficiency. In the case of glucose, *in vivo* ^1^H MRS detection is particularly challenging, as it exhibits a broad and complex spectral signature overlapped with the macromolecular baseline and with other main brain metabolites. Despite these challanges, glucose CEST (glucoCEST) imaging is undergoing important methodological developments^11,15^, with the use of unnatural glucose derivatives as biodegradable contrast agents^16^.

Glucose is considered the main source of energy for neurons^17^ and under normal conditions adenosine triphosphate (ATP) is almost exclusively produced by glucose oxidation^18^. While invasive measurements of glucose consumption can be done using specific micro-electrodes^19^ or post-mortem auto-radiography methods^17^, Positron Emission Tomography (PET) studies have reported minimally-invasive estimations of glucose’s cerebral metabolic rate (CMR) during brain activity^18,20,21^. Similar information has been revealed by fMRS techniques^22,23^. Compared to PET, MR based functional studies do not require radioactive tracers; they also present higher spatial and temporal resolutions, and can simultaneously produce high quality anatomical images^21^. In this paper, we explore CEST-MRI as a new method to probe metabolic changes occurring during brain activation. We present the first preclinical functional glucoCEST-fMRI results based on an acquisition method targeting the glucose CEST effects, which can be modulated by neuronal activation processes. Specifically, we detect a substantial change in the CEST properties of glucose, which could be related to a decrease in its accessibility. The latter can be linked to the consumption and thus the drop in its local concentration during neuronal activation. These findings are discussed and compared with existing literature.

## Results

### GlucoCEST-fMRI

**Figure 1:**
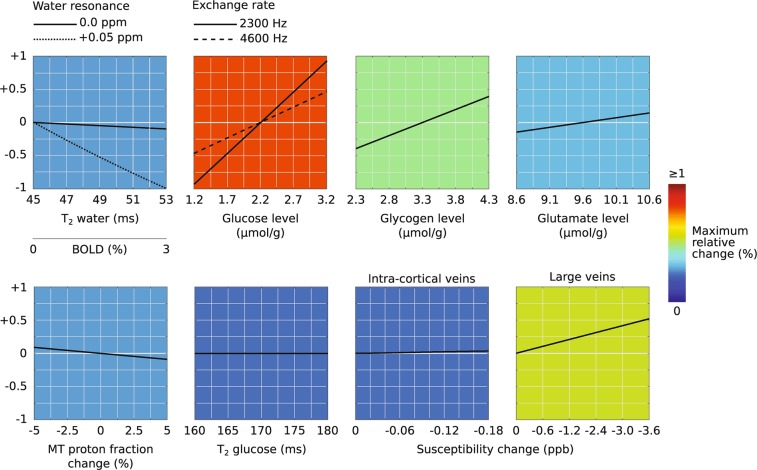
Functional CEST simulation results. The expected CEST-fMRI contrasts for the acquisition and reconstruction strategy adopted in this study, based on S(−1.2 ppm)/S(+1.2 ppm) signal ratio calculations plotted along the y-axis, are correlated against various potential variables: water T_2_*, glucose concentration, glycogen concentration, glutamate concentration, MT effects, glucose T_2_ and susceptibility changes. The colors were chosen according to the maximum expected relative changes (shown in %); note that a change in glucose’s exchange rate will also affect the obtained contrasts.

In order to design the optimal CEST-based fMRI protocol, numerical simulations based on Bloch-McConnell equations^24^ were performed. A six-pool CEST system (water, glucose, glutamate, glycogen, myo-inositol, NOE and magnetization transfer) was employed to test the robustness of several CEST functional acquisition and reconstruction approaches (see Supplementary Information and Fig. S2). The selected method, that we will refer to as CEST-fMRI, is based on the consecutive acquisition of images at symmetric CEST saturation frequencies +δ and −δ, followed by an image reconstruction of the spatially-dependent intensity ratios ${I}_{{\rm{ratio}}}(\delta )=\frac{I(-\delta )}{I(+\delta )}$ and a conventional fMRI analysis using Statistical Parametric Mapping (SPM). The simulations results presented in Fig. 1 show the expected CEST-fMRI contrasts for this approach, calculated and correlated against various potential variables –water T_2_^(^*^)^, glucose concentration, glycogen concentration, glutamate concentration, MT effect, glucose T_2_ and susceptibility changes. These calculations demonstrate that the ratio *I*_*ratio*_ (1.2 ppm) is optimally suited to highlight functional based contrasts (see Supplementary Information for details). We therefore used this approach to probe functional changes in the glucose CEST effects on rats subject to forepaw stimulation at 17.2 T.

**Figure 2:**
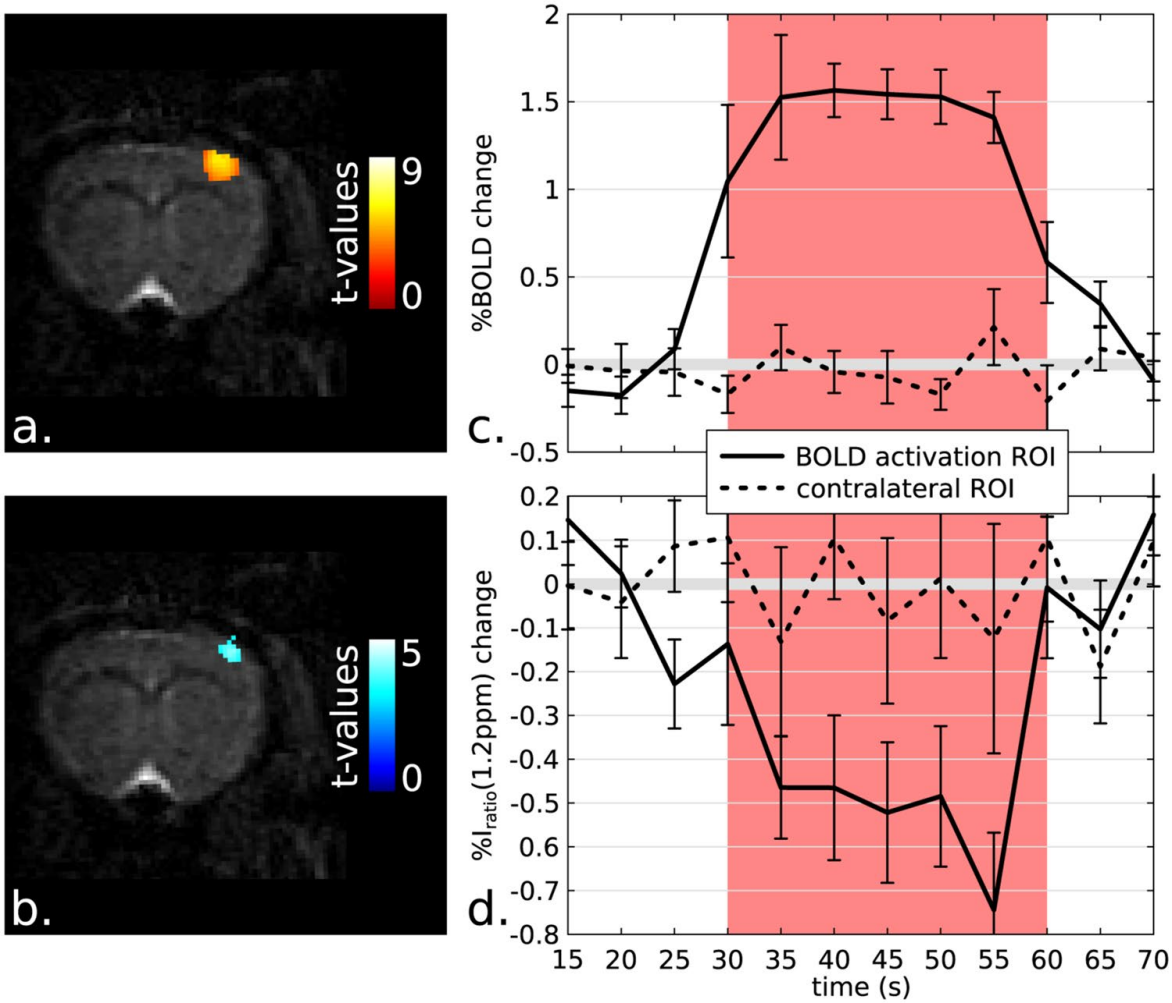
BOLD vs glucoCEST fMRI maps and time evolutions. Typical BOLD (**a**) and glucoCEST-fMRI maps (**b**) acquired on a rat subject to a 30 s on/off electrical forepaw stimulation at 17.2 T. Images were processed in SPM and displayed with a p-value threshold of 0.001. A local decrease of the image ratio I_ratio_(1.2 ppm) was detected during stimulation; notice its coincidence with the BOLD activation ROI. (**c**) BOLD and (**d**) glucoCEST-fMRI group mean time courses (n = 5 animals, 9 independent data sets). All time courses are showing relative changes compared to the baseline resting state (which was set at 0%). A substantial −0.5% relative decrease is observed for I_ratio_(1.2 ppm) during activation. As controls, time courses were extracted from the contra-lateral ROIs and these never revealed any significant changes (dashed lines). Note that the BOLD time evolution was extracted from the CEST-weighted images. The error bars represent the standard error of the mean (SEM) obtained over the n animals included in the group study.

Figure 2 shows representative outcomes of the glucoCEST and BOLD fMRI experiments. For all acquisitions, clear BOLD activation brain regions were detected in the somatosensory cortex areas (Fig. 2a). Remarkable contrast was also observed on the glucoCEST functional images acquired at δ = 1.2 ppm (Fig. 2b). Both activation maps highlighted similar brain locations, demonstrating that the detected glucoCEST contrast is linked with the brain activation mechanism. The average voxel number of the activation area was 90 ± 17 for BOLD and 40 ± 11 for the glucoCEST maps (n = 5 animals, 9 independent data sets). BOLD and glucoCEST time evolution signals extracted from the activation region of interest (ROI) are shown in Fig. 2c,d. The BOLD time evolution (Fig. 2c) was calculated using glucoCEST-weighted images; this resulted in an average BOLD effect lower than that reported in previous ultra-high field rodent fMRI^25^, due to the loss in time resolution (see Supplementary Information and Fig. S7). On the other hand, it permitted quality co-registration of simultaneously collected BOLD- and glucoCEST-based functional images. The average time course obtained for the glucoCEST signal (Fig. 2d) shows a substantial relative decrease of −0.5% for *I*_*ratio*_ during stimulation.

**Figure 3:**
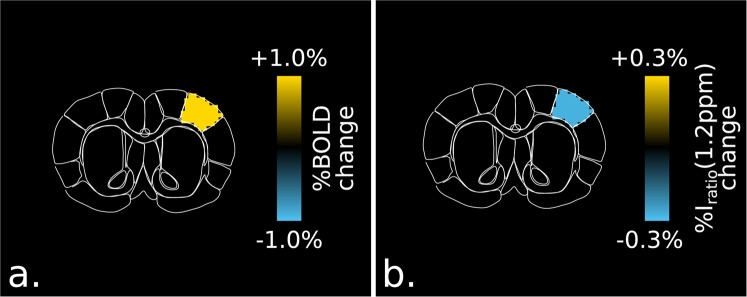
Segmented brain analysis of BOLD and CEST-weighted images. Segmented rat brain (Bregma +1.08 mm) showing the BOLD (**a**) and I_ratio_(1.2 ppm) contrasts (**b**) (p < 0.05, n = 5 animals, 9 independent data sets). An increase in BOLD and a relative decrease of the image ratio I_ratio_(1.2 ppm) are observable in the S1FL brain area.

In order to validate these observations, the BOLD and *I*_*ratio*_(1.2 ppm) time evolutions were extracted from the same contra-lateral ROIs; these never revealed any significant functional changes. Due to the relatively low temporal resolution of our protocol, the time-to-peak BOLD and glucoCEST responses were not compared. Figure 3 shows a brain segmented analysis of these BOLD- and glucoCEST-weighted data for n = 5 animals (identical data sets as for the previous analysis). Both positive BOLD and negative *I*_*ratio*_(1.2 ppm) contrasts were observed in the same primary somatosensory cortex of the forelimb area, S1FL, reinforcing the nature of the phenomenon being detected.

According to Fig. 1, the contrast observed is sensitive to changes in glucose concentration, and it depends on the glucose exchange rate as well as on changes in glycogen concentration if they exist. Taking into consideration these factors, we estimated that the −0.5% change in the CEST contrast observed experimentally would correspond to a minimum of −0.1 µmol/g change in glucose concentration (see Supplementary Information for details). This value does not reflect any absolute quantification but rather a lower bound estimation.

### Testing for possible confounds

In order to confirm that the observed glucoCEST-fMRI contrast at +1.2 ppm originates from chemical exchange effects between glucose and water, three additional sets of experiments were performed: functional spectroscopy to check for possible water frequency shifts, functional CEST imaging targeting 1.2 ppm but with low saturation power to check if the observed glucoCEST effect depends on B_1_, and functional CEST imaging targeting higher chemical shifts (3.5 ppm) to check for amide proton transfer (APT) and MT effect contaminations.

#### Water frequency shifts

**Figure 4:**
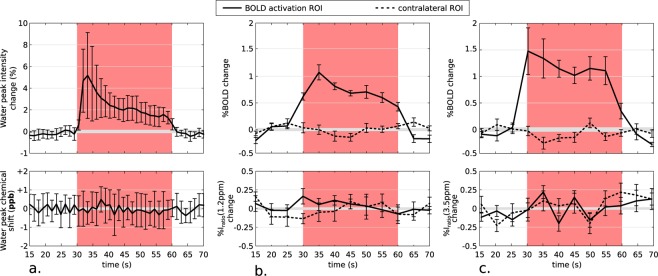
Results of the fMRS, low B_1_ CEST-fMRI and APT-fMRI experiments. (**a**) Group mean time courses of the water peak intensity (top) and the water chemical shift (bottom) obtained by analyzing fMRS data acquired from an 8-mm^3^ voxel centered on the BOLD activation region (n = 3 animals, 13 independent data sets). (**b**) Group mean time courses of BOLD (top) and glucoCEST-fMRI (bottom) signals acquired for a low B_1_ power (n = 5 animals, 8 independent data sets). (**c**) Group mean time courses of BOLD (top) and APT-fMRI (bottom) signals acquired at ± 3.5 ppm (n = 4 animals, 10 independent data sets). All time courses are showing relative changes compared to the baseline resting state (which was set at 0%). Average CEST-fMRI time courses were extracted for the activated ROI and for its non-activated contralateral counterpart, containing 65 voxels in average. While a significant BOLD effect was observed when using either fMRS (**a**, top) or CEST-fMRI (**b** and **c**, top), the water chemical shift remained constant around 0 ± 1 ppb (**a**, bottom) and no substantial changes were recorded for I_ratio_(1.2 ppm) at low B_1_ power (**b**, bottom) or for I_ratio_(3.5 ppm) (**c**, bottom). The error bars represent the SEM obtained over the n animals included in the group study.

Results of functional spectroscopy experiments are shown in Fig. 4a (n = 3 animals, 13 independent data sets). While a clear BOLD effect was observed (Fig. 4a, top), the analysis did not reveal any substantial variation of the water frequency during activation (Fig. 4a, bottom). This indicates that the water resonance frequency did not shift during the CEST-fMRI experiments, and therefore changes in overall susceptibility did not affect the glucoCEST contrast. These results are also supported by the analysis of the signal phase (see Supplementary Information and Fig. S8) which did not reveal any significant phase variations of the MR signal recorded in the activated ROIs.

In addition, CEST-fMRI experiments were performed for chemical shifts close to water such as 0.6 ppm (data not shown) and did not reveal any significant changes that could originate from transient Z-spectral shifts.

#### B_1_ dependency of glucoCEST-fMRI

CEST-fMRI experiments were performed on five animals using a single saturation RF pulse with a duration of 500 ms at a B_1_ field of 0.5  µT. Such low saturation power scheme was chosen to remove all possible sources of contrast due to CEST effects while keeping a substantial direct water saturation effect at 0 ppm. All other parameters were set following the glucoCEST-fMRI protocol described in the Methods section. BOLD and image ratio time evolutions are shown in Fig. 4b. Unlike the glucoCEST-fMRI results obtained for a B_1_ field of 3.5  µT shown in Fig. 2, no substantial relative changes was observed for I_ratio_(1.2 ppm) during activation (Fig. 4b, bottom). These findings confirm that the observed contrast recorded using glucoCEST-fMRI originates from B_1_-dependent chemical exchange effects.

#### APT/MT contamination

CEST-fMRI experiments were performed on four animals using an identical protocol as described for glucoCEST-fMRI but with focus on a higher chemical shift (±3.5 ppm). The aim was to determine if other broader signal contributions such as APT (at +3.5 ppm) or MT, which is maximum for −2 ppm^26^, could contribute to the glucoCEST-fMRI contrast. The resulting time series revealed no changes (Fig. 4c, bottom) and therefore no variations in the MT or APT effects upon stimulation. We therefore also conclude that these Z-spectral contributions remain constant during activation, and they do not contaminate the glucoCEST fMRI contrast.

### Dependency on temperature and pH

In order to evaluate the effect of changes in temperature and pH on the observed glucoCEST-fMRI contrast, *in vitro* experiments were performed at 17.2 T (see Supplementary Information for experimental details). The results obtained showed that a transient temperature increases due to brain activation can contribute slightly to the observed glucoCEST-fMRI contrast (a temperature increase of +0.1 °C would generate a drop of glucoCEST-fMRI contrast of less than 0.05%.). Changes in pH could also affect the observed contrast. Specifically, brain activation could potentially trigger a local pH drop leading to an increase of the glucoCEST-fMRI contrast, which is the opposite of what is observed in this manuscript.

### The effect of hyperoxia on the CEST-fMRI signal

**Figure 5:**
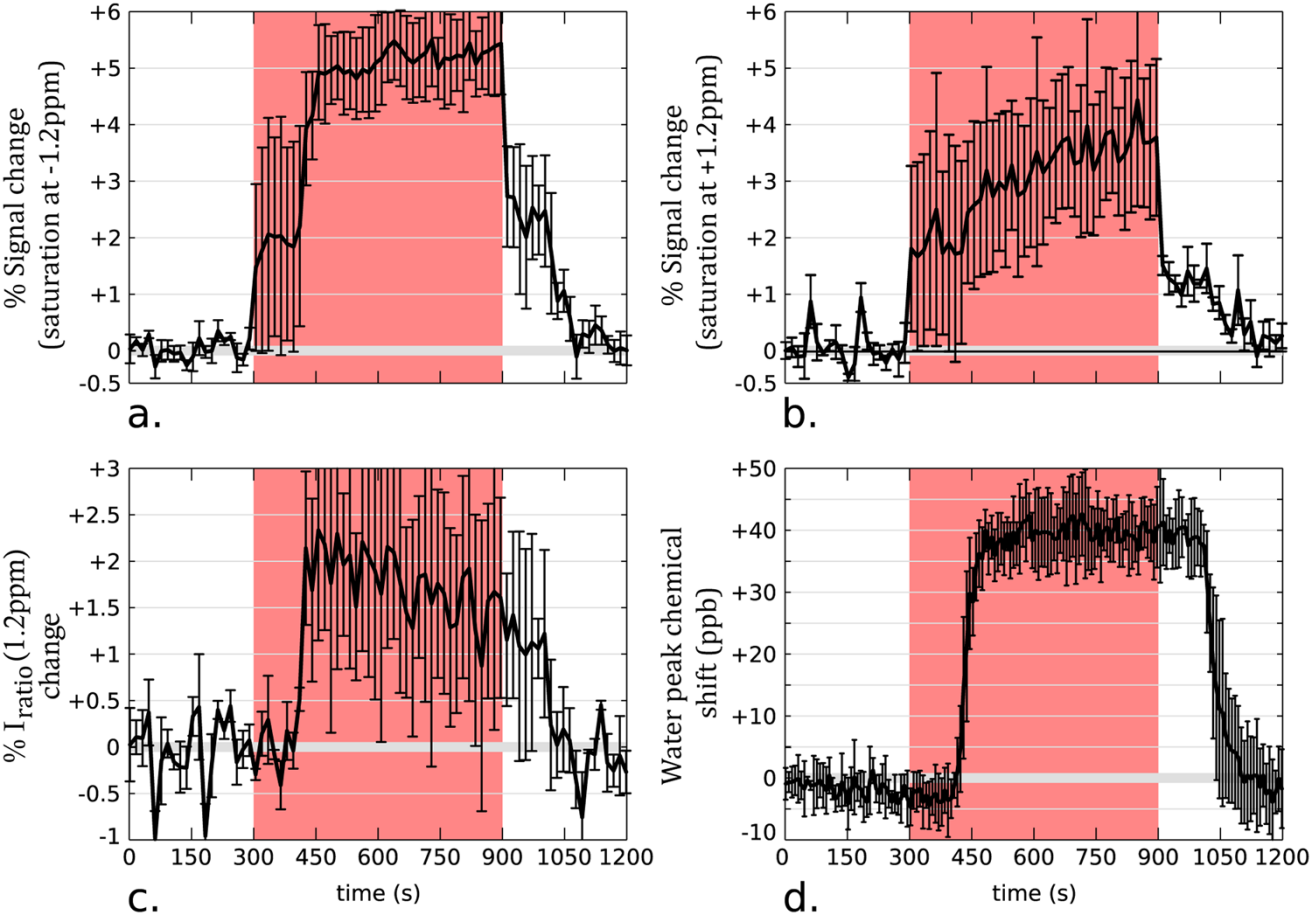
Results of glucoCEST-MRI and fMRS experiments upon hyperoxia. Group mean time courses of −1.2 ppm and +1.2 ppm CEST signals (**a,b**) and *I*_*ratio*_ (1.2 ppm) changes upon hyperoxia (**c**) (n = 3 animals, 3 independent data sets). (**d**) Group mean time courses of the water peak chemical shift obtained by analyzing fMRS data acquired from a 8-mm^3^ voxel (n = 2 animals, 5 independent data sets).

GlucoCEST-MRI and MRS data were acquired upon hyperoxia on three and two animals, respectively, using the protocols described in the Methods section. For the datasets with saturation at −1.2 ppm and +1.2 ppm, signals were extracted from an ROI covering the somatosensory cortex region and the corresponding time courses plotted in Fig. 5a,b, respectively. The time course of the ratio *I*_*ratio*_(1.2 ppm) displayed in Fig. 5c shows that hyperoxia has an effect on the acquired glucoCEST signal. An approximate +1.5% increase in *I*_*ratio*_(1.2 ppm) takes place during O_2_ administration, and it originates from the unequal signal changes occurring for saturations at −1.2 and +1.2 ppm. Indeed, the measured signal change upon hyperoxia was systematically higher when implementing CEST at −1.2 ppm than at +1.2 ppm resulting in a positive *I*_*ratio*_(1.2 ppm). This unbalance between upfield and downfield chemical shifts upon hyperoxia stems from a strong frequency shift of the Z-spectrum which was estimated to ca. +0.04 ppm using fMRS (Fig. 5d). Note that an identical fMRS experiment has been carried out to evaluate water frequency shifts due to susceptibility effects upon forepaw stimulation and did not reveal any shifts (Fig. 4a). While the susceptibility change caused by the saturation of the hemoglobin with oxygen is expected to induce a negative frequency shift^27^, during these hyperoxia experiments we observe a positive shift due to the presence of molecular oxygen (paramagnetic). Changes in the levels of brain glucose have been reported upon hyperoxia^28^; it is clear, however, that the *I*_*ratio*_(1.2 ppm) increase we observe upon hyperoxia is dominated by susceptibility effects that overpower such changes.

### Somatosensory cortex Z-spectrum

**Figure 6:**
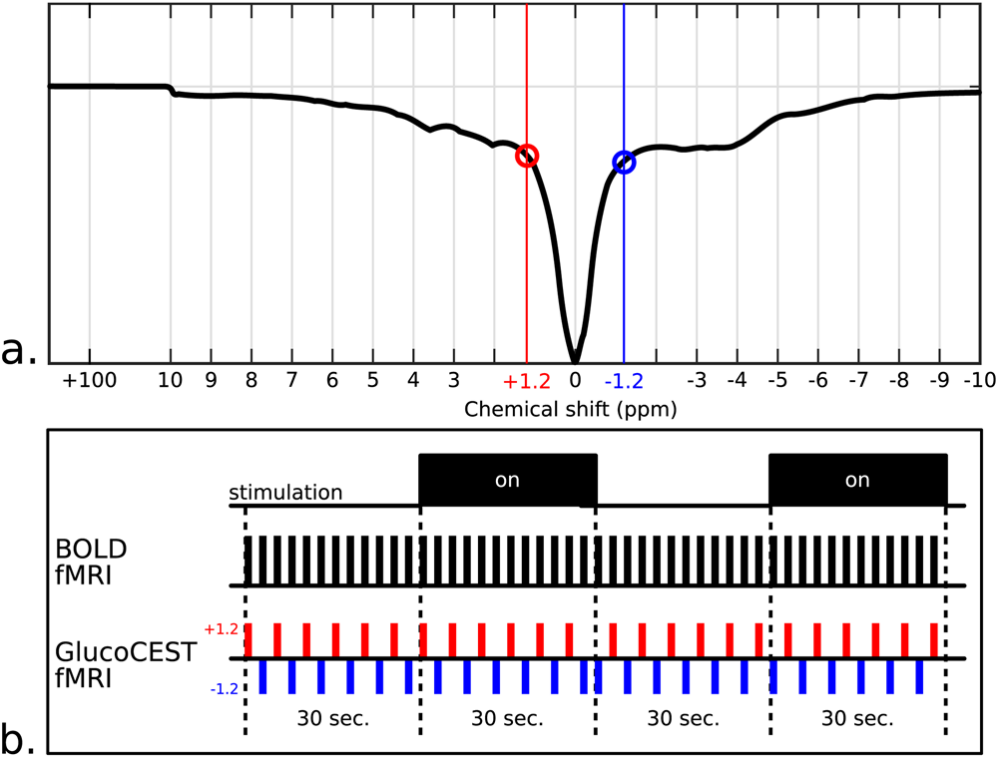
GlucoCEST-fMRI acquisition strategy. (**a**) *In vivo* Z-spectrum acquired from an 8-mm^3^ STEAM voxel placed in the somatosensory cortex of a rat brain (0.2-ppm step, 2 s/1.5 µT RF saturation). (**b**) Four-block timing sequences showing the stimulation paradigm, the conventional BOLD fMRI approach and the glucoCEST-fMRI data collecting strategy based on the consecutive acquisition of +1.2 ppm (red) and −1.2 ppm (blue) CEST images. Note the change in the acquisition order every two blocks in order to improve the BOLD compensation mechanism.

In order to initialize the numerical simulations (details available in the Supplementary Information), high resolution Z-spectral data has been acquired from an 8-mm^3^ STEAM voxel placed in the somatosensory cortex of a rat brain (0.2 ppm step, 2 s RF saturation at 1.5  µT). The obtained Z-spectrum, shown in Figs 6a and S1, shows clear exchange sites for various chemical shifts. APT effects are visible at +3.5 ppm, amines at ca. +2.7 ppm, labile amine sites of creatine and protein guanidino amine groups at ca +2 ppm^29^ and several broad and overlapped NOE and MT contributions on the upfield side of the Z-spectrum.

## Discussion

This paper presents the implementation of a CEST-based fMRI method, adapted to investigate neuronal activation. A negative glucoCEST contrast was observed upon performing electrical forepaw stimulations in rats at 17.2 T, when examining one of glucose’s labile resonances. Neuronal activation is a complex phenomenon which includes many neuro-chemical and biological changes^5,30^. Among those related to metabolism, changes in glucose concentration are most closely related to neuronal activation^17^, and hence this metabolite was targeted in the present study. Recent reports show that CEST imaging can be used to probe changes in glucose concentration by irradiating its hydroxyl resonances at +1.2 ppm^11,15,16,31,32^. On such basis this study introduces functional CEST-based measurements and explores their potential for real-time glucose metabolism detection. As a result, an interesting negative contrast matching in space and time the activation revealed by classical BOLD effects (Figs 2 and 3) was obtained. An average I_ratio_(1.2 ppm) relative decrease of -0.5% was measured during brain activation, highlighting a drop in the chemical exchange properties of glucose associated with neuronal activation. While glucose exchange rates are sensitive to micro-environmental parameters such as temperature and pH^15,31^, only few *in vivo* reports^33,34^ showed a local and limited temperature increase (+0.1 °C) and a slight pH drop^35^ as a result of brain activation. Such physiological parameters can potentially affect the measured CEST effect and intensity ratios. At 17.2 T, a +0.1 °C rise in temperature could potentially decrease the contrast by −0.05%, which remains a very low contribution (see Fig. S4). A drop in pH can nevertheless increase the contrast considerably (Fig. S5) but in the opposite direction than the functional results shown here. Moreover, Khlebnikov *et al*. recently confirmed that no pH changes could be detected during brain activation using an fMRI method based on APT^36^, known to be highly sensitive to pH and temperature^37,38^. In general, *in vivo* CEST-weighted experiments suffer from poor spectral selectivity because of the intrinsic nature of CEST (targeting inherently broad spectral resonances), but also because of the strong spectral overlapping between several assigned and non-assigned endogenous CEST agents^10^. GlycoCEST^39^ and MICEST^40^ imaging methods were recently developed to detect glycogen and myo-inositol, two major brain metabolites, by probing the [+0.5; +1.5] ppm CEST spectral region. Such spectral overlapping with glucoCEST measurements can potentially generate severe biases in this study. Neither fMRS nor non-MRI functional studies reported changes in myo-inositol levels during brain activation. Glycogen, however, is a key metabolite in the neuroactivation phenomena, and concentration changes have been successfully detected on animals using auto-radiography and micro-electrode measurements^41,42^ but not on humans^43^. The numerical simulations performed to study the mixing effect between glucose and glycogen (results provided in the Supplementary Information) reveal that changes in the glycogen concentration occurring during brain activation would contribute to the CEST contrast observed here.

In view of these considerations, we conclude that the glucoCEST-based fMRI contrast hereby observed originates mainly from a drop in the glucose and probably glycogen concentrations during neuronal activation. This finding is in agreement with previous fMRS studies, showing that the glucose consumption increases and therefore local glucose concentration momentarily drops during brain activation. Chen *et al*. measured a drop of 0.3  µmol/g in the glucose concentration of the human visual cortex during stimulation^8^, leading to a CMR of 0.512  µmol/g/min, in agreement with previous estimations^18^. Similar results were reported by Hyder *et al*. in the somatosensory cortex in rats, based on forepaw electrical stimulation paradigms akin to ours^9^. Recent clinical ^1^H fMRS and PET studies tend to agree on a basal glucose CMR of 0.4  µmol/g/min, with a 30% increase during visual stimulation and an estimate that brain glucose cannot decrease faster than ~0.1  µmol/g/min^5^. Using a more invasive technique based on glucose oxydase micro-electrodes, Silver *et al*. were able to measure local transient falls in glucose levels of 0.1 to 0.2  µmol/g during stimulation^19^. Finally, using similar methods, Li and Freeman report immediate decreases in extracellular glucose concentration of ~0.05  µmol/g during neural activation in cats^44^, for which the time courses closely matched neuronal spiking with glucose levels returning to baseline as neurons stopped firing. In summary and by taking into account a 30-s stimulation duration, a wide range of glucose concentration changes upon activation is available in the literature and ranges between −0.05 and −0.3 µmol/g. In agreement with these results, our glucoCEST-fMRI data also suggests a glucose concentration drop rapidly followed by an increase and a return to the original resting state level. The origins of these oscillations in glucose concentration are still debatable. Nasrallah *et al*. recently reported that D-glucose chemical exchange effects are originating mainly from the intracellular space^31^, while Chan *et al*. claim that most of the glucoCEST signals seen in tumors after injecting D-glucose are of extracellular origin^11^. The changes we observe could therefore be due to a decrease in both extra- and intra-cellular glucose concentration. Establishing the exact intracellular origin is of particular interest in neuroscience as both neurons and astrocytes are involved in the brain activation mechanisms^45^. With the addition of exogenous agents, glucoCEST-fMRI measurements of the kind hereby described may help pinpoint the origin of the reported changes.

Despite our reliance on ultra-high magnetic fields, the functional contrast of our experiments remains low, and required a substantial group study to obtain statistically significant results. It also required the careful design of a CEST-fMRI acquisition and processing strategy to quantify contributions that may mask the glucoCEST behavior being explored. Moreover, due to the poor selectivity of CEST and to the similarity in the exchange properties of glucose and glycogen, a non negligible part of the observed contrast can potentially originate from a glycogen concentration drop (see Supplementary Information). Additional experiments are necessary to disentangle the contribution of the two metabolites. It is conceivable that alternative quantification methods recently proposed for an enhanced robustness of CEST-MRI^46,47^, could also be incorporated with success into CEST-fMRI. Alternatively to CEST, the chemical exchange-sensitive spin-lock (CESL) MRI method, which is based on T_1_ρ measurements, is able to generate interesting functional contrast^48^ and was reported to detect glucose changes in cat brains during visual stimulation^16^. While the glucoCEST-fMRI protocol that was here introduced is devoid of potentially confounding effects associated to BOLD, T_2_^*^ (including T_2_) phenomena or MT effect, the method *per se* is not quantitative, as the image intensity I_ratio_(δ) is not normalized to any reference that can lead to absolute concentration changes. Still, refinements can be conceived that would also allow the *in vivo* quantification of these changes.

In general, B_0_ inhomogeneities can also affect conventional CEST-MRI results, and methods such as water saturation shift referencing (WASSR) are widely used to post-correct these heterogeneities^39^. The durations needed for such approaches are, however, incompatible with the speed requirements of stimulation-based functional measurements performed here. Instead, we ensured good field uniformities within the targeted brain regions using localized shimming procedures (Supplementary Information and Fig. S6). Frequency shifts originating from susceptibility changes can affect not only BOLD fMRI^27,49^ but also CEST-fMRI data, as evidenced by our simulations and hyperoxia experiments. By using the water frequency and linewidth changes between oxy- and deoxygenated blood, Song *et al*. suggested a method to filter out deoxygenated blood from the BOLD contrast by employing strong off-resonance RF pulses (530  µT for 8 s) for a range of chemical shifts between +0.8 and +3.5 ppm. While the CEST-fMRI presented here has similarities with Song’s method^27^, the purposes of the two methods are different and the RF pulse powers employed differ substantially. The changes in susceptibility and thus in water frequency shift are highly dependent on the type of vascularization (large vein or capillaries) and on the oxygenation and the cerebral blood volume (CBV) changes upon brain activation (see Supplementary Information). Bianciardi *et al*. suggested that while susceptibility changes are important in the case of large veins (where oxygenation increases but CBV remains constant), small intra-cortical veins show very small susceptibility changes likely because of the counter-balancing effect of a CBV increases in those type of vessels^49,50^. This seems to be the case in the current study as no water peak frequency shifts were detected upon forepaw stimulation using fMRS methods (Fig. 4a). Finally, no APT or MT changes were detected using CEST-fMRI at higher chemical shifts (Fig. 4b,c) confirming the recently published APT-fMRI findings^36^ and validating that such Z-spectral signals are not contributing to the glucoCEST-fMRI contrast.

To conclude, this study reports the development of a new fMRI tool to measure and image real-time metabolic changes upon neuronal activation. The ensuing CEST-fMRI method shows promising results, liable to further improvement and translation –along the lines of currently assayed glucoCEST experiments– into human investigations. Furthermore, CEST’s sensitivity advantage is not limited to glucose detection but could be also used to interrogate the metabolic pathways of other fundamental metabolites involved in neuronal activation such as glycogen and lactate. All these options are currently under investigation.

## Methods

### Animal preparation

26 male Sprague-Dawley rats (250–300 g, Janvier Labs, Saint-Berthevin, France) were used in this study. The rats were housed two per cage under controlled light conditions (7:00–19:00) and were given free access to water and food. All animal procedures used in the present study were approved by the *Comité d’Ethique en Expérimentation Animale, Commissariat à l’Energie Atomique et aux Énergies Alternatives, Direction des Sciences du Vivant (Fontenay-aux-Roses, France)* and by the *Ministère de l’Education Nationale, de l’Enseignement Supérieur et de la Recherche (France)* under reference APAFIS#4082-2016021510499450v2 and were conducted in strict accordance with the recommendations and guidelines of the European Union (Directive 2010/63/EU) and the French National Committee (Décret 2013–118). During all MRI exams the respiration rate and body temperature were monitored. The body temperature was maintained between 36.5 and 37 °C using heated water (Grant TC120, Grant Instruments, Shepreth, UK). To avoid motion-related artifacts the head was immobilized using a bite bar and ear pins.

### fMRI experiments

During the fMRI experiments, an air-oxygen gas mixture (33% oxygen in medical air) was delivered to a nose-cone mask for spontaneous respiration. The animals were anesthetized initially with 5% isoflurane, which was reduced to 2% for maintenance. After the insertion of the needle electrodes used for electrical forepaw stimulation, a bolus of 50 μg/kg medetomidine (Domitor®, Orion Pharma, Espoo, Finland) was injected subcutaneously, and the isoflurane level was progressively decreased and set to 0% approximately 10 min later. A continuous subcutaneous infusion of medetomidine (100 μg/kg/h) was started right after the bolus injection using an infusion pump (HARVARD PHD2000, Harvard Apparatus, Holliston, MA, USA). To perform the electrical forepaw stimulation, two pairs of needle electrodes (26 gauge) were inserted under the skin of the right and left forepaws (between digits 2 and 3 and digits 4 and 5). Either the right or the left paw was used for each fMRI acquisition. Electrical pulse stimulation was given with a constant current bipolar isolated pulse stimulator (model 2100; A-M Systems, WA, USA), triggered by a logic gate pulse from the Bruker imaging system. Rectangular pulses with 3-ms duration, 2-mA current and 10-Hz frequency (100-ms interval) were applied during 30 s (stimulation block), separated by a 30-s rest interval (rest block). GlucoCEST-fMRI experiments consisted of 12 blocks accounting for 6 min scan time. A 10-min delay was maintained between consecutive fMRI runs.

### Hyperoxia experiments

Each hyperoxia experiment lasted 20 minutes and consisted in delivering (i) an air-oxygen gas mixture (33% oxygen in medical air) during the first 5 minutes, (ii) a 100% O_2_ administration during 10 minutes and (iii) an air-oxygen mixture during the last 5 minutes. This experiment was repeated 4 or 5 times for each rat to increase sensitivity.

### Data acquisition design

Both glucoCEST and functional imaging require specific timing conditions for optimum performance. GlucoCEST-MRI necessitates a preparation period, where a continuous or discrete train of weak radiofrequency (RF) pulses with a certain offset relative to water adjusted according to the targeted chemical shift is applied. In order to increase the efficiency of the RF irradiation, this saturation duration is usually set between 2 and 5 s –depending on the repetition time (TR) and on the relaxation properties at a given field. By contrast, functional imaging usually requires a short TR in order to record the BOLD effect with the highest time resolution and functional contrast possible. In addition, when performing glucoCEST-fMRI experiments, one has to remove or compensate for any unwanted non-CEST effects such as BOLD/T_2_^(*)^, MT or susceptibility effects. In order to design an optimal glucoCEST-fMRI acquisition and reconstruction method capable of accommodating these dissimilar demands, numerical simulations were performed and are detailed in the Supplementary Information.

### GlucoCEST-fMRI

The pulse sequence used was based on an echo planar imaging (EPI) acquisition of alternate images with opposite CEST saturation frequencies, +1.2 and −1.2 ppm, using the block-design stimulation paradigm in Fig. 6b. As shown by the simulation results (see Supplementary Information), the image ratio, ${I}_{{\rm{ratio}}}(\delta )=\frac{I(-\delta )}{I(+\delta )}$, of these two consecutively acquired CEST-weighted images exhibits a glucoCEST contrast free of any unwanted contributions such as BOLD/T_2_^(^*^)^, MT or susceptibility effects. Since the BOLD effect is not constant during stimulation, the order of the CEST image acquisitions *I*(+*δ*) and *I*(−*δ*) was changed after each pair of stimulation blocks.

### MRI acquisitions

All experiments were performed on a 17.2 T horizontal scanner (Bruker BioSpin, Ettlingen, Germany) equipped with a gradient system allowing a maximum gradient strength of 1000 mT/m. A 30-mm diameter surface coil was used for RF transmission and reception. Animal positioning was performed using multi-slice fast low angle shot imaging (FLASH, TE/TR = 6/100 ms). Standard BOLD fMRI was performed using multi-slice GE-EPI (3 slices) in order to identify the BOLD activation area. Good field homogeneity was ensured using standard automatic iterative shimming followed by a FASTMAP^51^ on a volume of interest (VOI) containing the rat brain. After the FASTMAP, a 3D B_0_ map was acquired and a 2^nd^ order shimming was performed using MAPSHIM^52^ on a VOI placed on the slice of interest (c.a. 200 mm^3^). An example of a B_0_ map acquired after shimming is shown in Fig. S6. Common acquisition parameters for GE-EPI acquisitions were 2 × 2 cm Field Of View (FOV), 85 × 85 matrix size, 1.2 mm slice thickness, 450 kHz bandwidth, 50° flip angle, TE/TR = 9/2500 ms. The slice showing the strongest BOLD effect was selected for the single-slice glucoCEST-GE-EPI acquisitions. The CEST preparation consisted of a train of 40 50-ms rectangular pulses (10  µs inter-pulse delay, total saturation time = 2 sec) with a B_1_ of 3.5 µT and a saturation frequency offset alternating between +1.2 and −1.2 ppm, ending with a 0.2-ms gradient spoiler (2 G/mm).

### Data processing

The glucoCEST-fMRI reconstruction consists of a 3-stage processing pipeline which includes (i) a preprocessing of the data, (ii) a CEST asymmetry analysis, and (iii) the calculation of the activation maps using the SPM12 software (Wellcome Trust Centre for Neuroimaging, London, UK) and activation time courses. Stage (i) includes: manual image masking, voxel-by-voxel signal drift correction using a linear regression model, image registration using SPM, voxel-by-voxel time-domain spike removal (threshold set to 10% of average signal level) and image smoothing (kernel size of 7 voxels, σ = 4). Stage (ii) consists in calculating the glucoCEST-weighted images before performing the functional analysis. GlucoCEST-weighted images are calculated by generating image ratios ${I}_{{\rm{ratio}}}(\delta )$ for each pair of consecutive CEST images (Fig. 6b). In stage (iii) we used SPM to generate BOLD- and CEST-weighted activation maps. The time evolution of the glucoCEST-weighted signals was extracted from ROIs drawn on the BOLD activation maps. As a control, the same time evolution analysis was performed for a symmetric contra-lateral ROI of the brain. GlucoCEST-fMRI data was added to the group study analysis provided that SPM processed images showed significant (p < 0.05) contrast and that the BOLD signal change was higher than 0.5%. An additional functional analysis approach consisted in performing an image segmentation based on a rat brain atlas^53^ and averaging the voxel intensities within each brain ROI provided that the signal change was significant between rest and stimulated state (p < 0.05).

Functional spectroscopy experiments were performed in order to evaluate whether shifts in the water resonance frequency occur during neuronal activation. Spatially localized spectra of the water peak were acquired from an 8-mm^3^ voxel centered on the BOLD activation region. A STEAM sequence (TE/TR = 9/1500 ms) was employed using an identical stimulation paradigm, scan time and animal protocol as previously described. Each water spectrum was processed using 0^th^ and 1^st^ order phase corrections and evaluated in terms of water peak intensity and frequency. Time evolutions of these parameters were computed and was added to the group study analysis provided that the BOLD signal change observed on the water peak intensity was higher than 0.5%. Similar localized spectroscopy measurements were performed during hyperoxia.

The datasets generated during and/or analyzed during the current study are available from the corresponding author upon request.

## Supplementary information

Brain sugar consumption during neuronal activation detected by CEST functional MRI at ultra-high magnetic fields
