# Supplementary material for: Brain sugar consumption during neuronal activation detected by CEST functional MRI at ultra-high magnetic fields
Source: Sci Rep. 2019 Mar 14;9:4423. doi: 10.1038/s41598-019-40986-9 (PMC6418181; doi:10.1038/s41598-019-40986-9)
Supplement: Supplementary file 1 — Brain sugar consumption during neuronal activation detected by CEST functional MRI at ultra-high magnetic fields [file 41598_2019_40986_MOESM1_ESM.docx]

Supplementary Information

Brain sugar consumption during neuronal activation detected by CEST functional MRI at ultra-high magnetic fields

Tangi Roussel, Lucio Frydman, Denis Le Bihan and Luisa Ciobanu

# CEST-fMRI method design

**Functional Z-spectra simulation.** Numerical simulations of Z-spectra were performed in order to design the optimal CEST-fMRI signal acquisition and processing method and to evaluate whether it allows the detection of relative changes in glucose concentration while compensating for other sources of contrast. A six-pool system was modeled at 17.2 T using Bloch-McConnell equations1 and employing the Matlab tool “CEST sources” (available at [www.cest-sources.org](http://www.cest-sources.org/)). The model included several CEST agents (glucose, glutamate, glycogen, myo-inositol) as well as NOE and MT effects. Rest Z-spectra were simulated using the parameters listed in Table 1. The proton T_2_ of water, the proton fraction of NOE and MT effects were adjusted to visually fit the acquired *in vivo* data (Fig. S1); all other parameter values were taken from the literature.


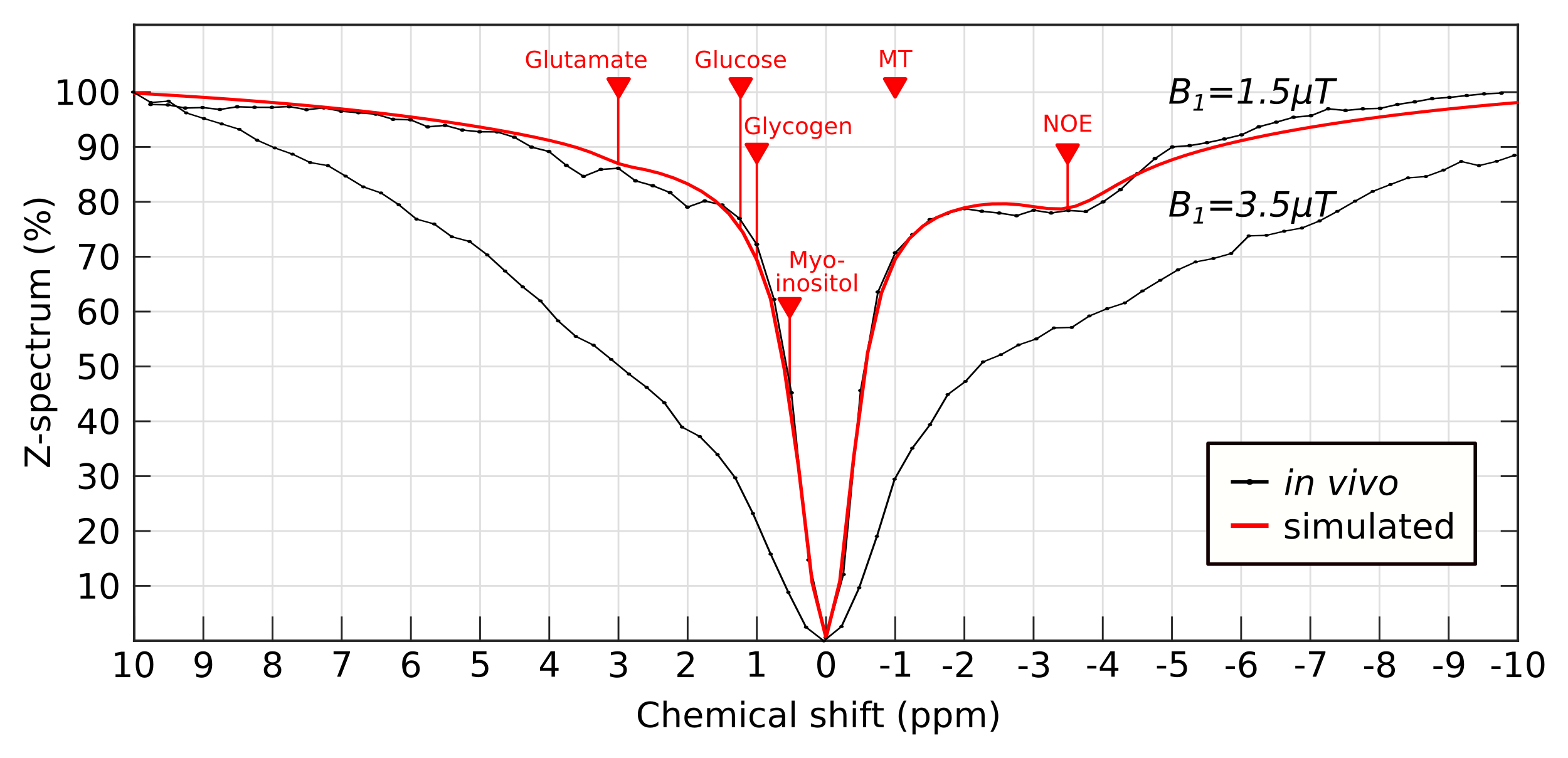


**Figure S1: Acquired and simulated Z spectra at rest.** (black) In vivo Z-spectra acquired from an 8-mm^3^ STEAM voxel placed in the somatosensory cortex of a rat brain; B_1_ power of 1.5 µT and 3.5 µT, RF saturation duration 2s, 0.2-ppm step. (red) Simulated Z-spectrum.

The Z-spectra obtained upon stimulation were simulated with an identical RF saturation scheme as employed during the experiments (B_1_=3.5 µT). Parameters known to be involved in the brain activation mechanisms were varied. The BOLD effect remains the main observable phenomena in fMRI and consists mainly in a change in the T_2_*/T_2_ of water. The T_2_ of water was increased from 45 to 53 ms mimicking a 0 to 3% BOLD effect. The T_2_ of glucose was varied within a ±10 ms range (between 160 and 180 ms), potentially affecting the exchange with water. The concentration of glucose, glutamate and glycogen were varied within a ±1 mmol/L range around their rest values mimicking metabolic level changes. The MT effect intensity was changed by varying its proton fraction within a ±5% range. Such asymmetric MT effects are known to distort the global lineshape of the Z-spectrum but remain very hypothetical in the context of brain activation. Finally, the water resonance frequency was shifted according to susceptibility changes which take place during brain activation and that have been reported in humans by Bianciardi *et al*.2 in large and intracortical veins.

|  | **Water** | **Glucose** | **Glutamate** | **Glycogen** | **Myo-inositol** | **NOE** | **MT** |
| --- | --- | --- | --- | --- | --- | --- | --- |
| **Concentration (mmol/L)** |  | 2.2 ^(^3) | 9.6 ^(^3) | 3.3 ^(^4) | 5.9 ^(^3) | 0.008 ^(*)^ | 5 ^(*)^ |
| **T_1_ (ms)** | 2100 | 1780 ^(^3) | 1699 ^(^3) | 1500 | 1608 ^(^3) | 1500 | 1000 ^(^1) |
| **T_2_ (ms)** | 45 | 170 ^(^3) | 79 ^(^3) | 100 | 170 ^(^3) | 1 | 0.05 |
| **Chemical shift (ppm)** | 0 and +0.05 | +1.2 ^(^5) | +3 ^(^6) | +1 ^(^7) | +0.6 ^(^8 ) | -3.5 ^(^9) | -1 ^(^10) |
| **Exchange rate (s^-1^)** |  | 2300-4600 ^(^12,13) | 2000 ^(^11) | 1000 ^(^7) | 600 ^(^8) | 10 ^(^9) | 20 |

**Table 1: Simulation parameters used to compute rest CEST spectra at 17.2 T.** Concentration values noted ^(^*^)^ are expressed in proton fraction values and were used to control NOE and MT effects.

Exchange rates are highly dependent on physiological parameters such as pH and the literature reports wide ranges of exchange rates for glucose12,13 going from 2300 to 4600 s^-1^. In order to evaluate the impact of such variability on our results, simulations were performed for a range of exchange rates between the two extreme values (Table. 1). Moreover, the impact of B_0_ shimming efficiency was determined by performing simulations for two shimming conditions where the water peak was on resonance (at 0.0 ppm) and slightly off-resonance (+0.05 ppm shift).

**CEST-fMRI processing approaches.** Six different processing approaches were designed involving different combinations of signal intensities at one, two or three Z-spectrum points, positioned at +1.2, -1.2 and +100 ppm respectively (Fig.5). For each approach, the functional contrast was estimated between rest and stimulated states. To evaluate the dependencies of each approach with respect to the various parameters and to find the optimal strategy to compensate all non-CEST contributions, the simulations were computed and analyzed separately for each approach, by comparing the change in the CEST-fMRI contrast with the input parameter change. For each input parameter and each approach, the maximum contrast (relative change) was evaluated and scaled on a blue-to-red colorbar.


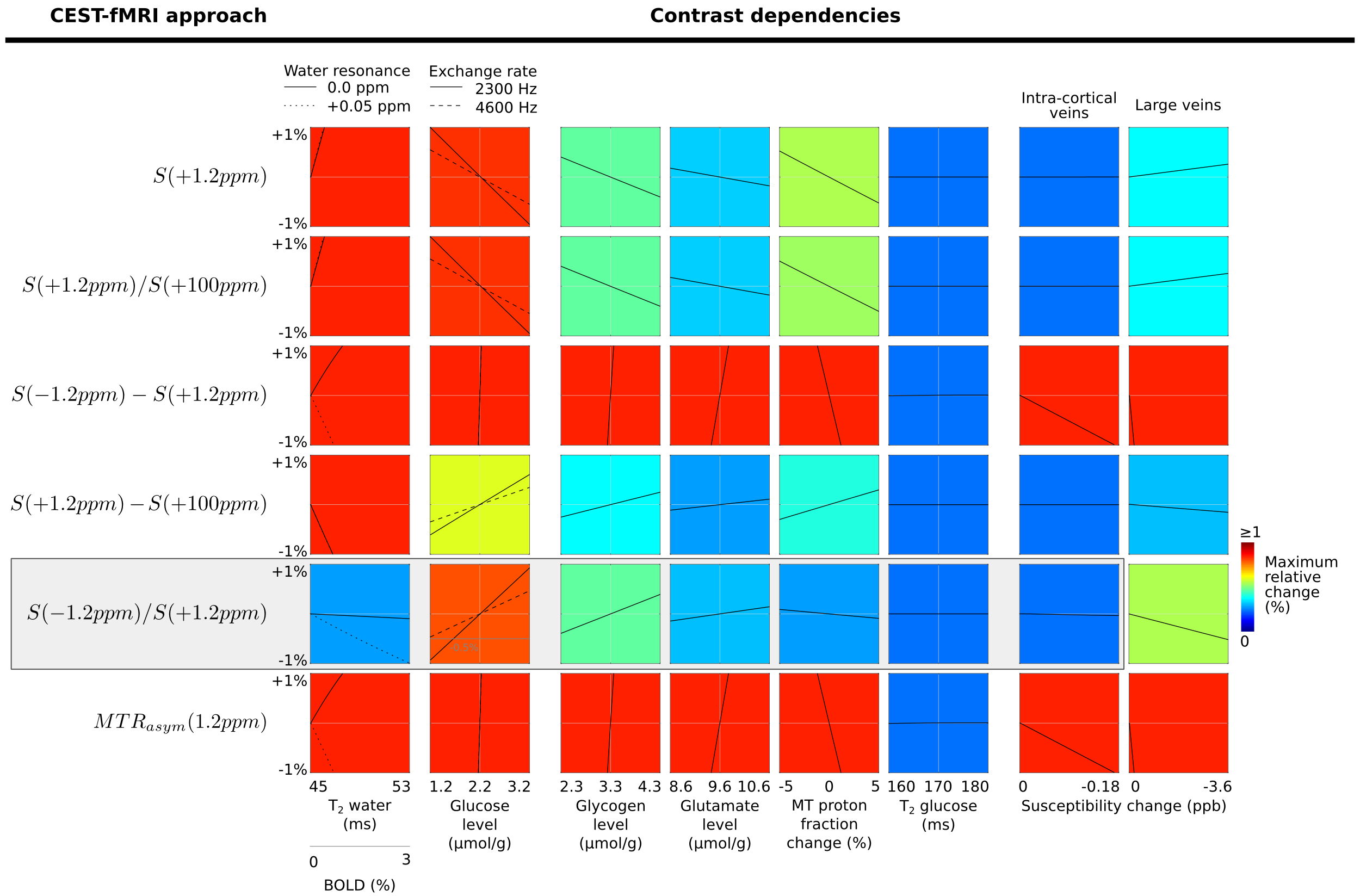


**Figure S2: Functional CEST simulation results.** CEST-fMRI contrasts calculated and correlated against various input parameters (water T_2_^(^*^)^, glucose concentration, glycogen concentration, glutamate concentration, MT effect, T_2_ of glucose and susceptibility change) for different CEST-fMRI acquisition and reconstruction strategies. The y-axes are expressed in percent (%) of relative changes and range between -1 and +1%. The plot color is scaled according to the maximum relative change observed. It can be noticed that a change in the glucose exchange rate has an impact on the obtained contrasts. In addition, water frequency shifts due to poor B_0_ shimming or susceptibility changes measured in a large vein during activation are severely contaminating the contrast for most of the approaches. In the case of intra-cortical veins, the effect is however negligible for some approaches. The approach highlighted by a gray box, which is based on the signal ratio calculation was found to most efficiently compensate BOLD, MT effects and susceptibility changes while generating fMRI contrasts that reflected the targeted change in glucose concentration. In this approach, glycogen level changes can also contribute to the measured signal and was taken into account in the following steps of the study.

The results of these evaluations are summarized in Fig. S2. The six different strategies produce a variety of functional contrasts depending on BOLD/T_2_^(^*^)^, MT, glucose concentration and susceptibility effects. For example, the simplest approach, which consists in measuring the signal change at +1.2 ppm during stimulation, will produce a contrast that is strongly dependent on BOLD and MT changes. This is not surprising, previous reports investigated the use of off resonance direct saturation pulses to exploit novel BOLD fMRI contrasts14. The approach with the least BOLD contamination is based on computing the ratio of signal intensities acquired for symmetric saturation frequencies [$S\left( -1\text{.}2\text{ppm} \right)/S\left( +1\text{.}2\text{ppm} \right)$]. In the case of a strong shift in the water frequency due to poor B_0_ shimming conditions or the presence of large veins (intense susceptibility change), this approach can show some limitations as seen in Fig. S2. However, the *in vivo* CEST-fMRI experiments presented here included a robust shimming protocol that resulted in high quality B_0_ field maps (see next section) and the targeted brain region is focused in the somatosensory cortex of the rat brain which mainly contains a dense capillary network15, with no water frequency shifts (Fig. 4a) or phase variations detected (Fig. S8).


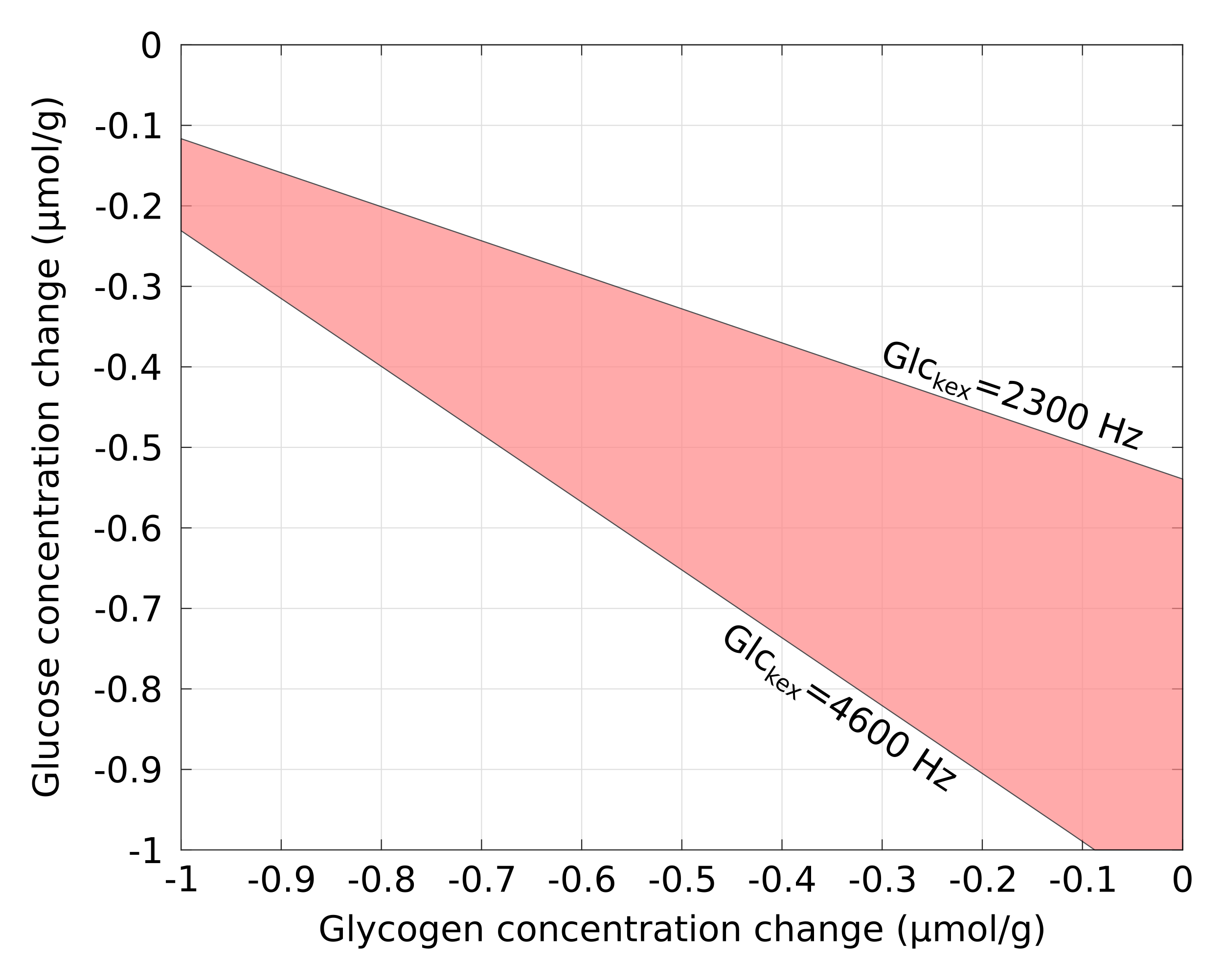


**Figure S3: Glucose and glycogen contributions to the -0.5% CEST-fMRI contrast.** The glucose concentration change was plotted versus the glycogen concentration change for a range of glucose exchange rates (Glc_kex_) of 2300 to 4600 Hz and a fixed glycogen exchange rate of 600 Hz. This plot was generated using the simulation results showed in Fig. S2 for a -0.5% drop of the ratio [$S\left( -1\text{.}2\text{ppm} \right)/S\left( +1\text{.}2\text{ppm} \right)$]. Both glucose and glycogen level changes can affect the glucoCEST-fMRI contrast depending on the glucose exchange rates.

**Glycogen contamination.** Calculating the ratio of symmetric saturation frequencies [$S\left( -1\text{.}2\text{ppm} \right)/S\left( +1\text{.}2\text{ppm} \right)$] constitutes therefore the optimal approach for CEST-fMRI. The simulation results show a strong correlation between the obtained contrast and the glucose concentration changes (Fig. S2). Strong changes in glutamate levels (±1 µmol/g) and the presence of 5.9 µmol/g of myo-inositol at +0.6 ppm did not affect significantly the CEST-fMRI contrast. On the other hand, a change in the glucose exchange rate or a change in the glycogen level can contribute to this contrast, as shown in Fig. S3. For an observed contrast of -0.5%, various combinations of glucose and glycogen drops can be estimated for a range of glucose chemical exchange rates (from 2300 to 4600 s^-1^). Glycogen is a key molecule in the brain activation metabolism but only few reports showed glycogen level variations during brain activation. In 1992, using autoradiography, Swanson *et al.* showed that lower levels of glycogen could be found in the somatosensory cortex of brain rats after forepaw stimulation16 but failed to perform any quantitative measurements. Using an *in situ* brain freezing technique17, Dienel *et al.* showed a glycogen level decrease of ca. -30% upon sensory stimulation in rats. The latter report also estimated a glycogen rest level of 12.6 µmol/g in disagreement with more recent reports4 in awake animals using ^13^C NMR, which established an average rest level of 3.3 µmol/g. Finally, in 2007, a ^13^C fMRS study on humans did not reveal any glycogen level change during visual cortex stimulation18. To our knowledge, no reliable *a priori* information about glycogen level drops during brain activation is available in the literature. As a precaution, we will however assume in this study a maximum glycogen drop of -30% which corresponds to ca. -1 µmol/g. Therefore, according to Fig. S3, the experimentally observed -0.5% CEST-fMRI contrast originates from a minimum glucose concentration change of -0.1 µmol/g. This value does not reflect any absolute quantification but rather a lower bound estimation.

# Temperature and pH dependencies

In order to evaluate the effect of changes in physiological parameters, such as temperature and pH, on the observed glucoCEST-fMRI contrast, *in vitr*o experiments were performed at 17.2 T. Samples of D-glucose (10 mmol/L) were prepared at three different pH values: 7.2, 7.4 and 7.6. For each sample, a Z-spectrum was acquired using a CEST-PRESS (Point RESolved Spectroscopy) sequence with an identical RF saturation scheme as employed during the experiments (B_1_=3.5 µT). For samples with pH 7.2 and 7.6, the temperature was maintained at 37°C while for the sample at pH=7.4 measurements were performed at various temperatures between 30 and 40°C. For each acquired Z-spectrum, the MTR_asym_ spectrum and the ratio [$S\left( -1\text{.}2\text{ppm} \right)/S\left( +1\text{.}2\text{ppm} \right)$] was calculated. For simplicity, the dependencies between the calculated CEST ratios and pH/temperature were assumed to be linear. While such assumption is probably incorrect over a large range of temperatures and pH values, it is acceptable for the small ranges of temperature (few degrees) and pH (<1 unit) studied here. The results relative to temperature and pH dependencies are shown in Figs. S4 and S5, respectively.

**
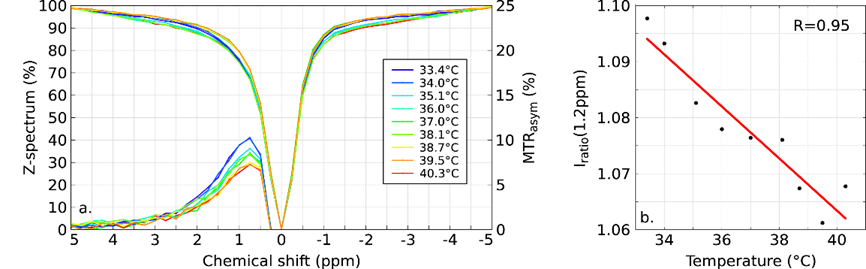
**

**Figure S4: GlucoCEST-fMRI contrast dependence on temperature.** In vitro glucose Z-spectra acquired at 17.2 T for various temperatures between 30 and 40°C (a), measured ratio [$S\left( -1\text{.}2\text{ppm} \right)/S\left( +1\text{.}2\text{ppm} \right)$] vs temperature (b). A potential temperature increase of 0.1°C induced by neuronal activation can lead to a glucoCEST-fMRI contrast drop of less than 0.05%.


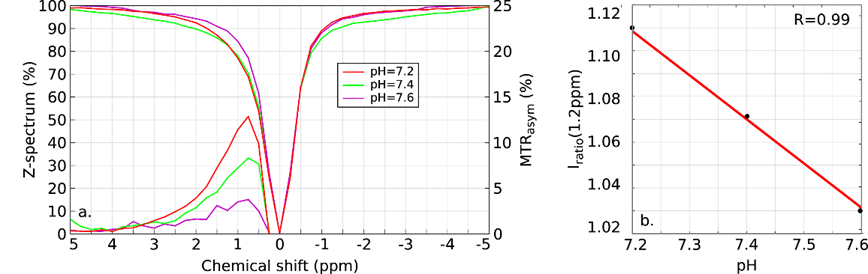


**Figure S5: GlucoCEST-fMRI contrast dependence on pH.** In vitro glucose Z-spectra acquired at 17.2 T for three pH values (7.2, 7.4 and 7.6) (a) and ratio [$S\left( -1\text{.}2\text{ppm} \right)/S\left( +1\text{.}2\text{ppm} \right)$] vs pH (b). A pH decrease will increase the glucoCEST contrast.

As suggested by the literature relative to CEST imaging19,20, the glucoCEST-fMRI contrast depends significantly on physiological parameters such as temperature and pH. Trübel *et al.* estimated a local average temperature increase of +0.1°C due to neuro-activation in forepaw stimulated rats21. Fig. S4 suggests than such temperature change could generate a drop of glucoCEST-fMRI contrast of less than 0.05%. Therefore, the transient temperature increases due to brain activation can contribute slightly to the observed glucoCEST-fMRI contrast (ca. 10% of the signal change observed experimentally). The *in vitro* results also showed that the glucoCEST-fMRI contrast depends significantly on pH: a pH decrease of 0.2 units increased the ratio [$S\left( -1\text{.}2\text{ppm} \right)/S\left( +1\text{.}2\text{ppm} \right)$] of ca. 4%. However, very few studies have shown significant changes in pH during neuro-activation. Magnotta *et al.* reported ca. -0.15% relative pH drops in the human visual cortex upon visual stimulation22 while Khlebnikov et al. did not report any changes using APT-fMRI23. Quantitative and absolute pH measurements are needed to accurately determine the impact of pH on glucoCEST-fMRI results. Such measurements could be performed for example with ^31^P MR spectroscopic methods24. To conclude on pH effects, brain activation could potentially trigger a local pH drop leading to an increase of the glucoCEST-fMRI contrast, which is the opposite of what is observed in this manuscript.

# Evaluation of B_0_ inhomogeneities


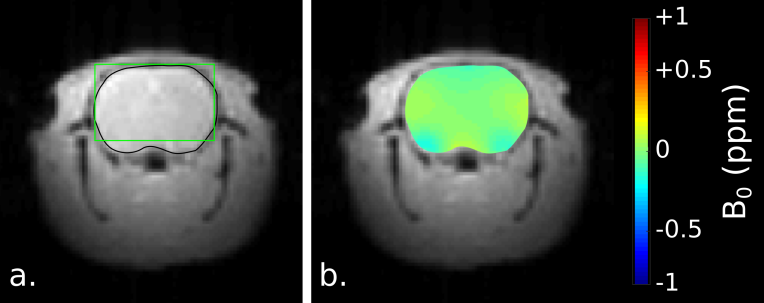


**Figure S6: Representative B_0_ field map.** Anatomical (a) and B_0_ field map (b) obtained on a rat brain at 17.2 T. The values represented in the B_0_ map are expressed in ppm. The green ROI in a. represents the VOI for which the field homogeneity was optimized.

As described in Methods section, good field homogeneity was ensured using standard automatic iterative shimming followed by a FASTMAP adjustment25 on a volume of interest (VOI) containing the rat brain. After FASTMAP, a 3D B_0_ map was acquired and 2^nd^ order shimming was performed using the MAPSHIM correction26 on a VOI placed on the slice of interest (c.a. 200 mm^3^). An example of B_0_ map acquired after the shimming procedure was performed is shown in Fig. S6.

# BOLD versus CEST-weighted BOLD

The average BOLD effect measured in this study is lower than that reported in previous ultra-high field rodent fMRI27. Fig. S7 compares the time courses of the relative BOLD change for the same rat using conventional GE-EPI BOLD imaging (TR=1500 ms, 10 blocks) and CEST-fMRI (TR=2500 ms, 12 blocks, see method section for other parameters) for δ=+1.2 and -1.2 ppm. A decrease of the BOLD effect is observed and originates from the lower time resolution used for the CEST-fMRI. As the BOLD response varies substantially in time, an increase in TR from 1.5 to 2.5 s increases the sampling time, which in turn (a) reduces the temporal SNR, increases the signal variability and (b) potentially omits some key time points especially at the initial phase of the brain activation.


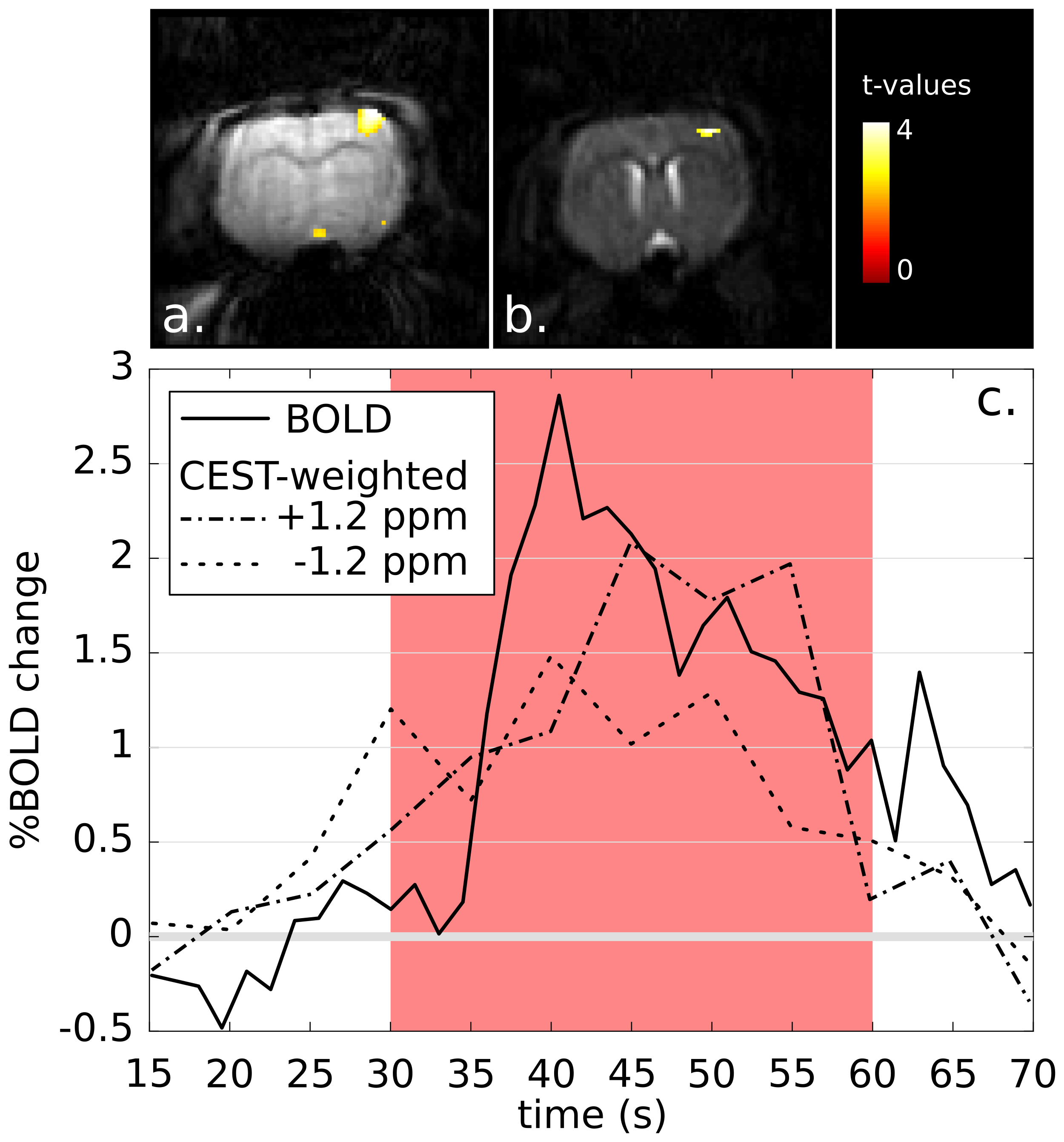


**Figure S7: BOLD versus CEST-weighted BOLD.** BOLD activation map obtained from a conventional GE-EPI BOLD experiment (a), CEST-weighted BOLD activation map (saturation at +1.2 ppm) obtained from a CEST-fMRI experiment on the same rat (b). Time courses were extracted and compared (c). A global drop in the BOLD effect is observed when using CEST at either +1.2 or -1.2 ppm because of the loss in time resolution.

# Evaluation of phase variation

The time evolution of the signal phase was also followed in order to evaluate whether shifts in the water resonance frequency occur during neuronal activation. The phase information was extracted from the BOLD imaging scans (not from the CEST-weighted ones), for both the activated and the contra-lateral ROIs. The GE-EPI images were reconstructed in the phase domain; an fMRI preprocessing was performed (see stage (i) in Method section) followed by a phase unwrapping in the time domain, plus a zeroth and first order phase correction to remove any experimental drift in time. Phase variations due to the respiratory cycle were removed by frequency filtering. Results of the phase analysis of the resulting fMRI signal are shown in Fig. S8. While a clear BOLD effect is observed (2% average peak), the analysis did not reveal any significant phase variations of the MR signal recorded in the activated ROIs (n=5 animals, 8 independent data sets). This indicates that the water resonance frequency did not change during the CEST-fMRI experiments; therefore, changes in overall susceptibility did not affect the glucoCEST contrast.


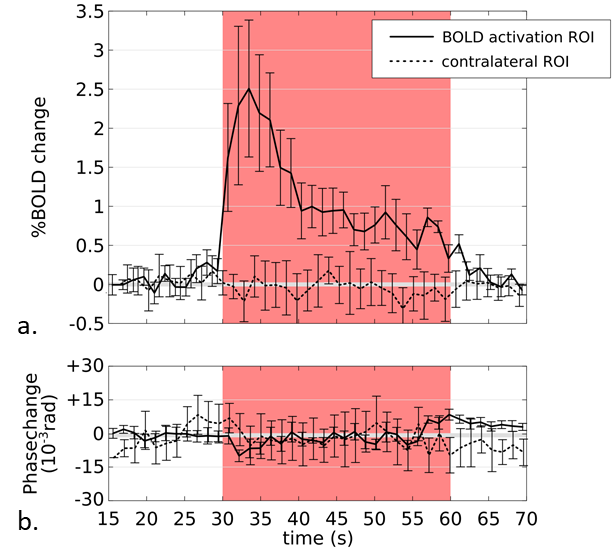


**Figure S8: Time evolution of phase during fMRI scans.** Average time courses were extracted for the activated ROI and for its non-activated contralateral counterpart, containing 65 voxels in average. (a) BOLD signal time evolution and (b) phase time evolution, both extracted from the multi-slice GE-EPI images (n=5 animals, 8 independent data sets). While a significant BOLD effect is observed during activation, no significant changes in the phase of the signal were detected. As controls, time courses were extracted from the contra-lateral ROIs (dotted line). The error bars represent the SEM obtained over the n animals included in the group study.

## **References**

1. Zaiss, M. et al. A combined analytical solution for chemical exchange saturation transfer and semi-solid magnetization transfer. *NMR Biomed.* **28**, 217-230 (2015).

2. Bianciardi, M.; van Gelderen, P. and Duyn, J. H. Investigation of BOLD fMRI resonance frequency shifts and quantitative susceptibility changes at 7 T. *Hum. Brain Mapp.* **35**, 2191-2205 (2014).

3. Lopez-Kolkovsky, A. L.; Mériaux, S. and Boumezbeur, F. Metabolite and macromolecule T1 and T2 relaxation times in the rat brain in vivo at 17.2T. *Magn. Reson. Med.* **75**, 503-514 (2016).

4. Choi, I.-Y. and Gruetter, R. In vivo 13C NMR assessment of brain glycogen concentration and turnover in the awake rat. *Neurochem. Int.* **43**, 317-322 (2003).

5. Chan, K. W. Y. et al. Natural D-glucose as a biodegradable MRI contrast agent for detecting cancer. *Magn. Reson. Med.* **68**, 1764-1773 (2012).

6. Haris, M. et al. Imaging of glutamate neurotransmitter alterations in Alzheimer's disease. *NMR Biomed.* **26**, 386-391 (2013).

7. McMahon, M. T.; Gilad, A. A.; Bulte, J. W. and van Zijl, P. C. Chemical Exchange Saturation Transfer Imaging: Advances and Applications. (eds. McMahon, M. T.; Gilad, A. A.; Bulte, J. W. and van Zijl, P. C.) (Pan Stanford 2017).

8. Haris, M.; Cai, K.; Singh, A.; Hariharan, H. and Reddy, R. In vivo mapping of brain myo-inositol. *Neuroimage* **54**, 2079-2085 (2011).

9. Geades, N. et al. Quantitative analysis of the z-spectrum using a numerically simulated look-up table: Application to the healthy human brain at 7T. *Magn. Reson. Med.* **78**, 645-655 (2017).

10. Swanson, S. and Pang, Y. MT is Symmetric but Shifted with Respect to Water. *Proc. Intl. Soc. Mag. Reson. Med. 11*. Toronto, Canada (2003).

11. Cai, K. et al. Magnetic resonance imaging of glutamate. *Nat. Med.* **18**, 302-306 (2012).

12. Jin, T.; Mehrens, H.; Hendrich, K. S. and Kim, S.-G. Mapping brain glucose uptake with chemical exchange-sensitive spin-lock magnetic resonance imaging. *J. Cereb. Blood Flow Metab.* **34**, 1402-1410 (2014).

13. Yadav, N. N. et al. Natural D-glucose as a biodegradable MRI relaxation agent. *Magn. Reson. Med.* **72**, 823-828 (2014).

14. Donahue, M.; Hua, J.; Edden, R.; Smith, S. and van Zijl, P. Detecting Brain Activity Using Direct Water Saturation. *ISMRM*. (2008).

15. Gagnon, L. et al. Quantifying the microvascular origin of BOLD-fMRI from first principles with two-photon microscopy and an oxygen-sensitive nanoprobe. *J. Neurosci.* **35**, 3663-3675 (2015).

16. Swanson, R. A.; Morton, M. M.; Sagar, S. M. and Sharp, F. R. Sensory stimulation induces local cerebral glycogenolysis: demonstration by autoradiography. *Neuroscience* **51**, 451-461 (1992).

17. Dienel, G. A.; Wang, R. Y. and Cruz, N. F. Generalized sensory stimulation of conscious rats increases labeling of oxidative pathways of glucose metabolism when the brain glucose-oxygen uptake ratio rises. *J. Cereb. Blood Flow Metab.* **22**, 1490-1502 (2002).

18. Oz, G. et al. Human brain glycogen content and metabolism: implications on its role in brain energy metabolism. *Am. J. Physiol.* **292**, E946-E951 (2007).

19. van Zijl, P. C. M. and Yadav, N. N. Chemical exchange saturation transfer (CEST): what is in a name and what isn't?. *Magn. Reson. Med.* **65**, 927-948 (2011).

20. Harris, R. J. et al. pH-weighted molecular imaging of gliomas using amine chemical exchange saturation transfer MRI. *Neuro Oncol* **17**, 1514-1524 (2015).

21. Trübel, H. K. F.; Sacolick, L. I. and Hyder, F. Regional temperature changes in the brain during somatosensory stimulation. *J. Cereb. Blood Flow Metab.* **26**, 68-78 (2006).

22. Magnotta, V. A. et al. Detecting activity-evoked pH changes in human brain. *Proc. Natl. Acad. Sci. U.S.A.* **109**, 8270-8273 (2012).

23. Khlebnikov, V. et al. Establishing upper limits on neuronal activity-evoked pH changes with APT-CEST MRI at 7 T. *Magnetic resonance in medicine* **80**, 126-136 (2018).

24. Deutz, N. E.; Bovée, W. M. and Chamuleau, R. A. Brain 31P NMR spectroscopy in the conscious rat. *J. Neurosci. Methods*. **16**, 151-161 (1986).

25. Gruetter, R. Automatic, localized in vivo adjustment of all first- and second-order shim coils. *Magn. Reson. Med.* **29**, 804-811 (1993).

26. Kanayama, S.; Kuhara, S. and Satoh, K. In vivo rapid magnetic field measurement and shimming using single scan differential phase mapping. *Magn. Reson. Med.* **36**, 637-642 (1996).

27. Ciobanu, L. et al. fMRI contrast at high and ultrahigh magnetic fields: insight from complementary methods. *Neuroimage* **113**, 37-43 (2015).
